# Beyond the freedom to refuse patient: A retrospective comparative study of emergency transportation during the COVID-19 pandemic in Japan

**DOI:** 10.1371/journal.pone.0331535

**Published:** 2026-07-09

**Authors:** Kaiho Hirata, Takuyo Chiba, Reo Takaku, Chen Meilai, Shunya Ikeda, Takashi Shiga

**Affiliations:** 1 Department of Emergency Medicine, International University of Health and Welfare Narita Hospital, Chiba, Japan; 2 Department of Emergency Medicine, AdventHealth Tampa, Tampa, Florida, United States of America; 3 Graduate School of Economics, Hitotsubashi University, Tokyo, Japan; 4 Department of Public Health, International University of Health and Welfare, Chiba, Japan; Medical Park Minatomirai, JAPAN

## Abstract

Emergency department overcrowding and ambulance diversion are significant problems worldwide and have become more apparent during the COVID-19 pandemic. In Japan, the Dedicated Emergency Physician (DEP) model has been associated with reduced transport time. However, whether this benefit persists during the pandemic remains unclear. This study analyzed the changes in transport outcomes during the COVID-19 pandemic in Japanese regions with and without DEP hospitals to evaluate the effectiveness of the DEP model and identify factors that could improve transport outcomes. Using nationwide data from January 2015 to December 2021, three target areas were analyzed: Urayasu-Ichikawa (DEP Group 1), Shonan-Fujisawa (DEP Group 2), and Ichinomiya-Toyota (Non-DEP Group 3). DEP Groups 1 and 2 contained DEP hospitals, while Non-DEP Group 3 was selected for its comparable population size and strong pre-pandemic transport performance. To minimize the impact of regional variations in COVID-19 prevalence, this study compared the changes in transportation outcomes before and after the pandemic between the target areas and nearby comparison areas. In total, there were 150,856 transports in Group 1; 186,965 in Group 2; and 516,655 in Group 3. In the target areas of Groups 2 and 3, transport time changes were significantly shorter by 2.016 and 0.606 min, respectively, compared to the comparison areas. Moreover, these areas had significantly lower odds of transportation difficulty (Group 2: odds ratio 0.131, 95% confidence interval 0.110–0.157; Group 3: OR 0.086, 95% CI 0.066–0.112). It was found that the common characteristics of these areas were densely located large-scale hospitals and makeshift buildings for patients with COVID-19, adjacent to large-scale hospitals. These findings suggest that DEP hospitals alone did not guarantee favorable transport outcomes during the pandemic. A sufficient number of large-scale hospitals and nearby temporary facilities may be crucial for maintaining effective emergency transportation during a pandemic.

## Introduction

Emergency department (ED) overcrowding and ambulance diversion have long been recognized as important problems that lead to increased morbidity and mortality [1–3], which will potentially worsen for decades owing to the rapid aging of developed countries [4,5]. Although several studies have proposed solutions, these problems have not been resolved [6–8]. Furthermore, during the COVID-19 pandemic, ED overcrowding and ambulance diversion have become more apparent, mainly because of the increased rate of patients with high-acuity conditions and patient management changes, leading to prolonged lengths of stay in the ED [9–11].

ED overcrowding problem has been an important issue in Japan for several decades. Transportation time in Japan has become longer, and one of the reasons is the unique emergency medical service (EMS) system, where each hospital has the discretion to decide whether to accept or reject an EMS request for patient transportation [12]. When the EMS is activated, paramedics assess the patient’s condition and identify the appropriate hospital. Patient transportation is initiated only after acceptance is obtained from the hospital. If a hospital declines acceptance, the paramedics contact another hospital. Hospitals have no legal obligation to accept emergency patients. Based on this “freedom to accept/reject” system, the EMS rejection by the hospital is a widespread phenomenon in Japan. Furthermore, the number of difficult hospital acceptance cases, defined as EMS rejections of >3 times, has increased dramatically since the onset of the COVID-19 outbreak in 2020, according to the Ministry of Health, Labour and Welfare [13]. In 2022, a quarter of the patients who were severely ill were rejected more than once when they called an ambulance, with the maximum number of rejections being 272 [14].

In Japan, EDs can be divided into two distinct models: the critical care and Dedicated Emergency Physician (DEP) models [15]. The critical care model focuses primarily on tertiary-level, patients who are critically ill. In this model, emergency physicians traditionally manage only patients who are critically ill, whereas most emergency care is delivered by on-call physicians from various specialties, many of whom have limited formal training in emergency medicine. In contrast, the DEP model is characterized by emergency physicians who dedicate themselves exclusively to emergency care and work in shift-based schedules. These physicians provide care to all emergency patients regardless of age or disease severity, and are responsible for the initial assessment, diagnosis, treatment, and patient disposition. Although this model has been adopted widely in North America, its implementation in Japan remains limited. Consequently, DEP-based emergency care is only available in selected institutions and regions.

The number of EMS rejections was low in areas with a DEP Model and was decreased when the government increased reimbursements for emergency care [15, 16]. Particularly, the DEP Model, which is similar to the emergency room (ER) system in the U.S., was shown to have a significatn impact on pre-hospital transportation time, reducing it by approximately 7 min [15]. However, whether this model contributes to the positive effects during the COVID-19 pandemic and the factors that could improve transport outcomes remain understudied.

To address this knowledge gap, we present a retrospective study examining the changes in transportation outcomes before and after the COVID-19 pandemic in areas with and without the DEP model. Comprehensive emergency transportation data from January 2015 to December 2021 were used in this study. This study aimed to examine whether the DEP system remains associated with favorable outcomes and whether other factors contribute to favorable outcomes.

## Materials and methods

### Study design

This was a retrospective observational study based on a Japanese national database from January 1, 2015, to December 31, 2021. All patients transported to hospitals by ambulance in Japan, except in Tokyo, were registered. Both ground and air transport were included. The database included the time course of transportation, age, sex, location of call, reason for transport, and severity (death, severe, moderate and mild). The severity was determined by the physician in charge at the time of hospital arrival. Severe and moderate patients are those who are expected to be hospitalized for ≥3 weeks and for <3 weeks, respectively. Mild patients are those who expected not to require hospitalization.

Two regions were selected as the DEP model target areas based on the previous research (DEP Group 1: Urayasu City and Ichikawa City in Chiba Prefecture; and DEP Group 2: Kamakura City, Chigasaki City, Fujisawa City and Zushi City in Kanagawa Prefecture) [15]. These areas are known for the presence of DEP hospitals (DEP Group 1: Tokyo Bay Urayasu Ichikawa Medical Center; and DEP Group 2: Shonan Kamakura General Hospital), which are defined as hospitals with more than 15 DEP physicians, including senior staff and senior residents. Among the five major metropolitan areas in Japan, Aichi Prefecture (population of 7.4 million) was selected because it has a comparable population size to Chiba Prefecture (population of 6.2 million) and Kanagawa Prefecture (population of 9.2 million), and it also faces the sea. Within Aichi Prefecture, one region was selected as a Non-DEP model target area based on its favorable emergency transport performance before the COVID-19 pandemic (Non-DEP Group 3: Ichinomiya City, Seto City, Kasugai City, Tsushima City, Toyota City and Inuyama City in Aichi Prefecture).

For each area, this study selected a nearby area for comparison, which was geographically adjacent to the selected area and comparable in terms of population, size, and location. The comparison areas were Funabashi City for DEP Group 1; Odawara City, Isehara City, Hatano City, and Sagamihara City for DEP Group 2; and Nagoya City for Non-DEP Group 3. Patients with missing data or transportation reasons other than medical illnesses were excluded.

In Chiba Prefecture, which has a population of 6.2 million, one of the top five hospitals accepting emergency transport received approximately 7,000 patients per year. Therefore, this study defined a large-scale hospital as one that accepts over 7,000 ambulances per year and calculated the Herfindahl-Hirschman Index of transportation to large-scale hospitals in each area. This was calculated based on the Hospital Function Report in 2022 [17].

This study was approved by the Ethics Committee of the International University of Health and Welfare, Narita Hospital (24-Im-014, June 25, 2024). Informed consent was not required to conduct this study because it was an observational study without intervention, and the data did not include identifiable personal information.

### Study outcomes

The primary outcomes were transportation time, defined as time from arrival at the scene to arrival at the hospital and the number of transportation difficulty cases, defined as a case with ≥4 phone calls by EMS to hospitals until the patient is accepted [18].

### Statistical analysis

To minimize regional disparities in COVID-19 spread, the changes in transportation performance were compared between the target and comparison areas in each group based on the assumption that geographically adjacent regions with similar population sizes and characteristics would be affected similarly by the COVID-19 pandemic. This approach likely reduced, although not eliminated, bias due to regional variation in the pandemic impact.

A multivariate analysis was conducted to compare the changes in transportation time and the number of transportation difficulty cases before and after the COVID-19 pandemic in the target and comparison areas. These changes were evaluated using the interaction term of a binary variable for the target areas and a binary variable for the COVID-19 period, similar to difference-in-difference analysis. The COVID-19 period is defined as the period from January 2020, when the first COVID-19 outbreak occurred in Japan, until December 2021. Because the trends in transport outcomes diverged between the comparison group and the target group due to the impact of COVID-19 even before the pandemic peak in 2021, we defined the treatment period as January 2020 onward and compare it against the pre-COVID-19 period (December 2019 and earlier), in order to capture the average effect across the pandemic as a whole.

In the multivariate analysis, we controlled for the time from emergency call to arrival at the scene, number of emergency calls within the same hour in the same EMS department, location of call, severity (death, severe, and moderate), and basic patient characteristics such as age and sex. The fixed effects for the EMS department and years/month were also controlled for. Standard errors were clustered at the EMS department level to address serial correlations within each EMS department [19]. A stratified analysis of the transportation time and difficulty was also conducted. Stratification factors included respiratory disease, oxygenation, and severity (severe, moderate, and mild).

Since pre-hospital transportation time was a continuous variable, this study used ordinary least squares to implement a multivariate analysis. Similarly, a logistic regression analysis was used for transportation difficulty because it is a binary variable. For each outcome, stratified analyses were also performed based on following categories: whether the primary reason for transport was respiratory diseases, oxygen supply during transport, and the severity (severe, moderate, and mild). Stata (version 18.0; Stat Corp, College Station, TX, USA) was used for statistical analysis, and a two-tailed P value of < 0.05 was considered statistically significant.

## Results

**Fig 1:**
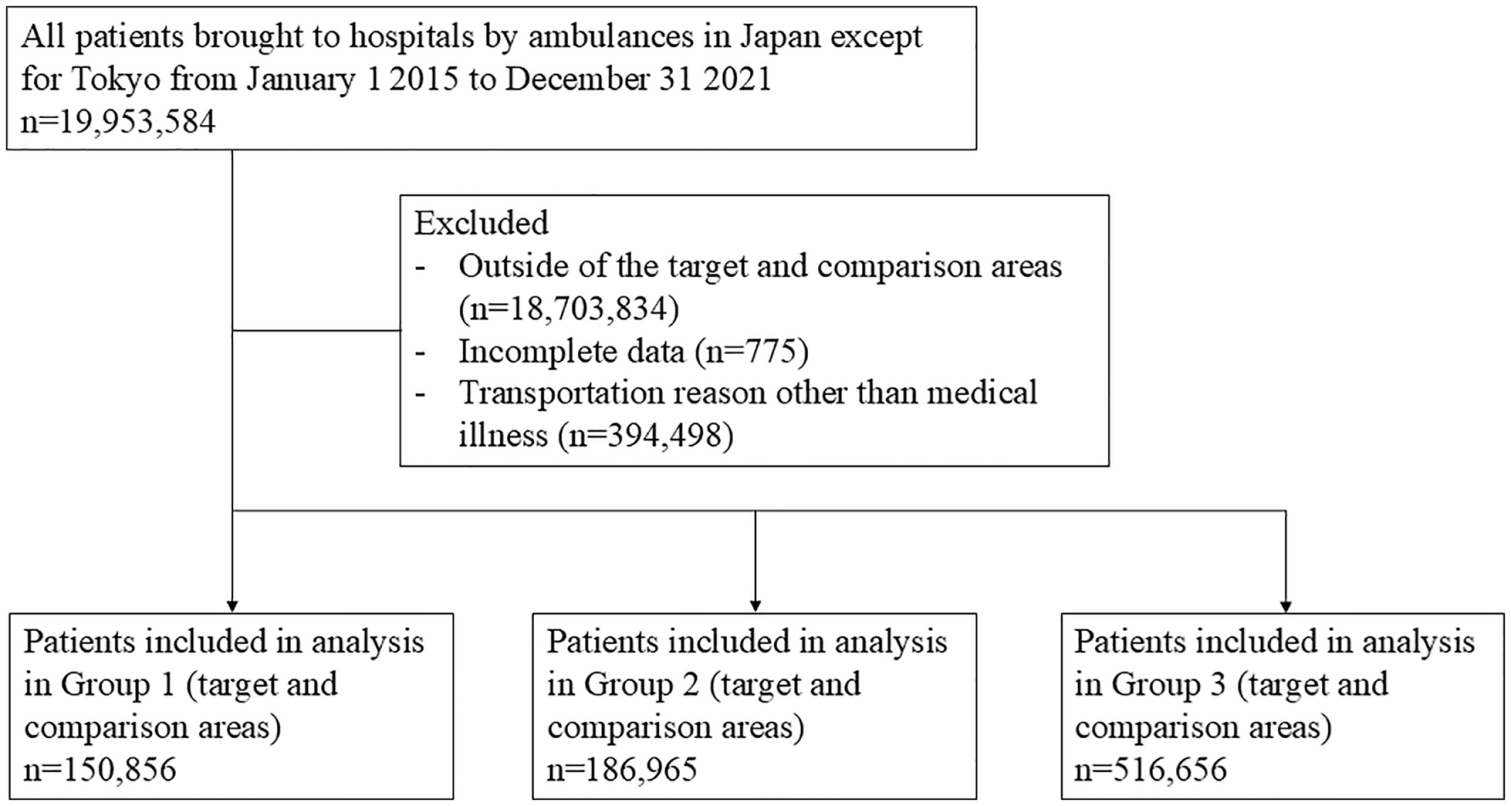
Patient enrolment.

In total, 19,953,584 patients were included in this study. After excluding patients outside the target and comparison areas, those with incomplete data or transportation reasons other than medical illnesses were excluded; 150,856 patients in Group 1, 186,965 in Group 2, and 516,656 in Group 3 were included in the analysis (Fig 1).

The background information of each group is summarized in Table 1. While the total number of transportations was similar between the target and comparison areas, the share received by large-scale hospitals (those accepting over 7,000 ambulances per year) varied considerably: 31% in Group 1, 68% in Group 2, and 80% in Group 3. In terms of demographic characteristics, the proportion of older adults was quite similar between the target and comparison groups, suggesting that the demand for ambulance use was comparable. The baseline characteristics of patients in each group are presented in Table 2. The mean time from call to hospital was shortest in Group 3 target area, and the percentage of transportation difficulty cases was shortest in Group 3 target area.

**Table 1:** Background characteristics of each group.

	Group 1 – Chiba		Group 2 – Kanagawa		Group 3 – Aichi	
	Target area	Comparison area	Target area	Comparison area	Target area	Comparison area
Hospitals accepting ambulances	11	9	17	14	21	52
Large-scale hospitals	1	0	3	0	8	6
Annual transportations	32913	23146	48127	34777	78730	120346
Annual transportations per hospitals accepting ambulances	2992	2572	2831	2484	3749	2314
The percentage of transportations to large-scale hospitals in whole transportations (%)	31	0	68	0	80	46
Herfindahl-Hirschman Index of transportations to large-scale hospitals	1956	1467	1782	1213	904	537
Population	668038	642907	909064	1178568	1642196	2332176
Percentage of people older than 65 (%)	21	24	27	28	26	25

Note: The definition of large-scale hospital is a hospital which accepts more than 7,000 ambulances per year.

**Table 2:** Patient characteristics.

	Group 1 – Chiba		Group 2 – Kanagawa		Group 3 – Aichi	
	Target area	Comparison area	Target area	Comparison area	Target area	Comparison area
**Observations**	73,481	77,375	118,274	68,691	197,920	318,736
**Time from emergency call to the hospital, mean (min)**	41	46	33	40	29	31
**Time from emergency call to the scene, mean (min)**	8	9	7	8	7	6
**Number of calls within the same hour**	2	3	2	2	2	11
**Age, median (IQR)**	67(40-81)	72(47-83)	75(52-84)	74(53-83)	74(52-83)	71(47-83)
**Age group, n (%)**						
0-9	4,454(6.06)	3,832(4.95)	5,624(4.76)	2,695(3.92)	8,918(4.51)	13,525(4.24)
10-19	2,366(3.22)	2,080(2.69)	3,115(2.63)	1,937(2.82)	5,609(2.83)	8,421(2.64)
20-29	6,148(8.37)	5,068(6.55)	5,554(4.70)	3,363(4.90)	8,610(4.35)	23,489(7.37)
30-39	5,256(7.15)	4,251(5.49)	5,238(4.43)	2,957(4.30)	8,848(4.47)	17,706(5.56)
40-49	6,115(8.32)	5,665(7.32)	7,735(6.54)	4,439(6.46)	13,156(6.65)	23,374(7.33)
50-59	6,980(9.50)	6,329(8.18)	9,567(8.09)	5,594(8.14)	15,517(7.84)	27,735(8.70)
60-69	7,815(10.64)	7,439(9.61)	11,281(9.54)	7,431(10.82)	20,814(10.52)	34,337(10.77)
70-79	14,104(19.19)	17,143(22.16)	23,925(20.23)	15,654(22.79)	46,957(23.73)	65,176(20.45)
80-89	15,148(20.61)	19,103(24.60)	33,246(28.11)	17,842(25.97)	52,434(26.49)	78,298(24.57)
90+	5,095(6.93)	6,465(8.36)	12,989(10.98)	6,779(9.87)	17,057(8.62)	26,675(8.37)
**Female sex, n (%)**	36,251(49.33)	38,399(49.63)	59,967(50.70)	33,149(48.26)	93,602(47.29)	152,823(47.95)
**Location, n (%)**						
Home	54,604(74.31)	57,033(73.71)	88,641(74.95)	51,060(74.33)	149,540(75.56)	224,602(70.47)
**Transport reason, n (%)**						
Brain disease	3,625(4.93)	3,978(5.14)	4,679(3.96)	4,405(6.41)	11,928(6.03)	17,377(5.45)
Cardiovascular disease	3,540(4.82)	4,472(5.78)	9,896(8.37)	4,686(6.82)	16,054(8.11)	24,697(7.75)
Gastrointestinal disease	6,015(8.19)	5,547(7.17)	7,628(6.45)	4,427(6.44)	14,541(7.35)	32,291(10.13)
Respiratory disease	6,029(8.20)	7,425(9.60)	8,331(7.04)	5,309(7.73)	17,326(8.75)	33,310(10.45)
Psychiatric disease	1,528(2.08)	2,168(2.80)	2,688(2.27)	1,805(2.63)	5,024(2.54)	8,852(2.78)
Neurological disease	1,329(1.81)	1,009(1.30)	3,018(2.55)	1,934(2.82)	11,057(5.59)	12,311(3.86)
Urologic disease	1,991(2.71)	2,031(2.62)	2,977(2.52)	1,918(2.79)	7,170(3.62)	10,877(3.41)
Neoplastic disease	591(0.80)	1,391(1.80)	952(0.80)	992(1.44)	3,434(1.74)	4,406(1.38)
Others	29,504(40.15)	8,263(10.68)	47,504(40.16)	9,265(13.49)	27,719(14.01)	35,689(11.20)
Unclear	19,329(26.30)	41,091(53.11)	30,601(25.87)	33,950(49.42)	83,667(42.27)	138,926(43.59)
**Oxygen supply during transportation, n(%)**	11,024(15.00)	12,673(16.38)	23,774(20.10)	15,266(22.22)	43,966(22.21)	64,062(20.10)
**Severity, n (%)**						
Death	419(0.57)	398(0.51)	2,060(1.74)	1,543(2.25)	3,858(1.95)	1,470(0.46)
Severe	4,460(6.07)	4,810(6.22)	8,946(7.56)	5,975(8.55)	14,728(7.44)	10,963(3.44)
Moderate	32,842(44.69)	36,436(47.09)	66,246(56.01)	34,801(50.66)	84,586(42.74)	124,704(39.12)
Mild	35,760(48.67)	35,731(46.18)	41,022(34.68)	26,472(38.54)	94,748(47.87)	181,599(56.97)
**Transportation difficulty cases, n (%)**	2,753(3.75)	3,563(4.60)	181(0.15)	2,206(3.21)	121(0.06)	4,842(1.52)

**Fig 2:**
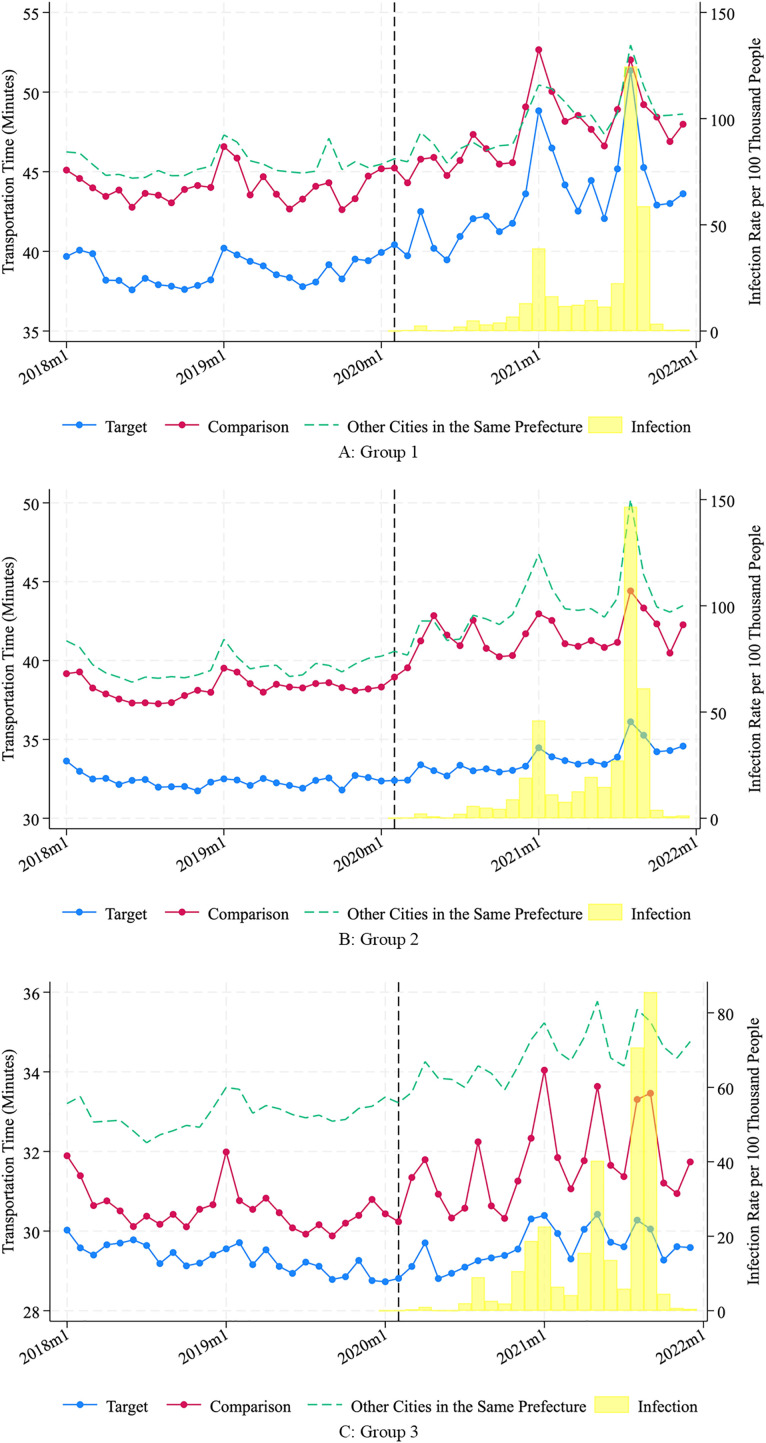
Trends of transportation time and infection rates per 100,000 populations in each area.

**Fig 3:**
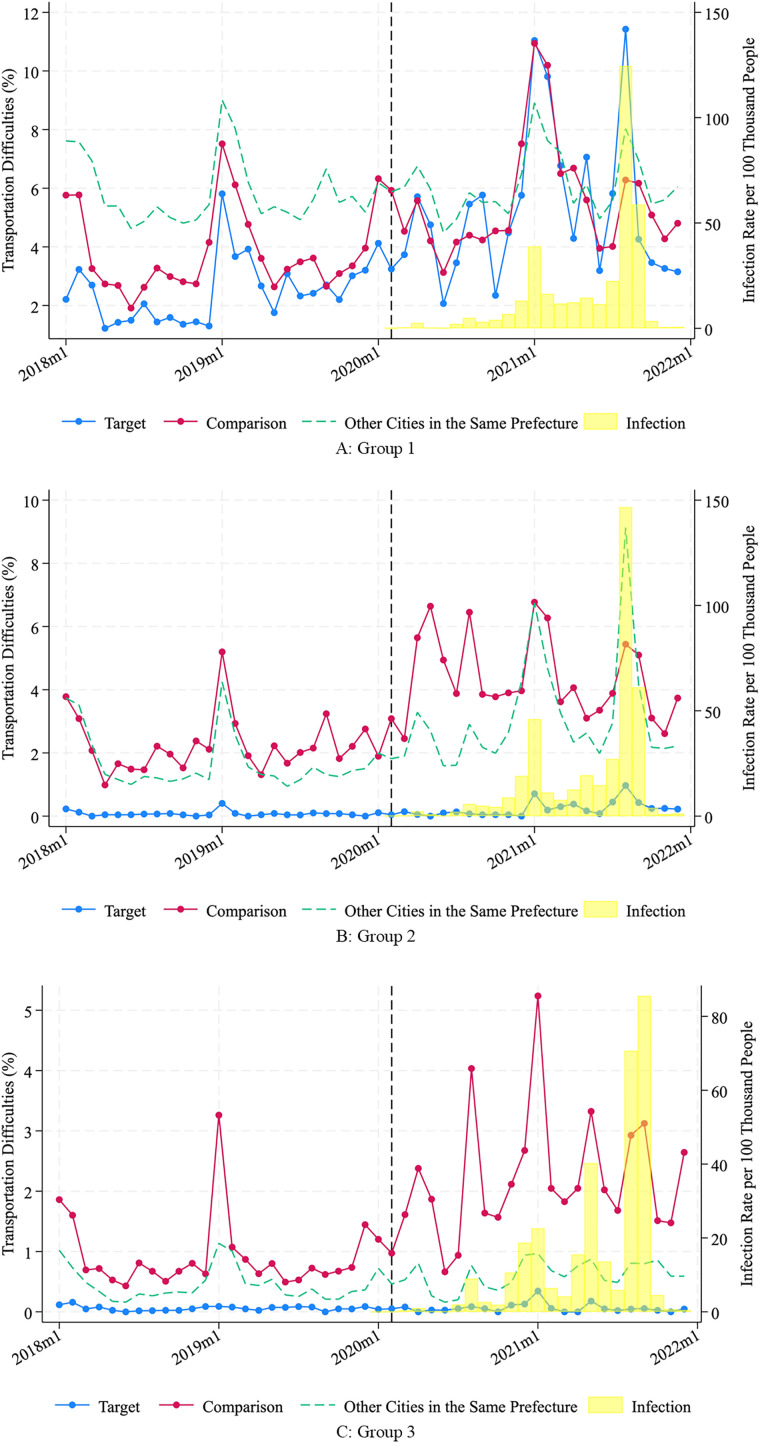
Trends of transportation difficulty cases and infection rates per 100,000 populations in each area.

The raw data trends for the transportation time are shown in Fig 2. Graphically, transportation time increased in the target area of Group 1 in a manner similar to that of the comparison area. Although the comparison area showed an increasing trend, the target area showed an unchanged trend during the pandemic in Group 2. In Group 3, there was an increasing trend in the target area; however, this trend was less prominent than that in the comparison area. Fig 3 shows the trends in the number of cases of transportation difficulty. Although the target area in Group 1 showed an increasing trend, similar to that of the comparison area, the target areas in Groups 2 and 3 showed unchanged trends during the pandemic. The results of the multivariate regression and stratified analyses of changes in transportation time and transportation difficulty are summarized in Table 3. In Groups 2 and 3 target areas, the relative changes in transportation time compared with the comparison areas were −2.016 min (95% confidence interval [CI] −3.631 to −0.400) and −0.606 min (95% CI −1.114 to −0.098), respectively. The odds ratios of transportation difficulty in the target areas compared to the comparison areas in Groups 2 and 3 were 0.131 (95% CI 0.010–0.157) and 0.086 (95% CI 0.066–0.112), respectively. This result was observed even after stratification by patients with respiratory diseases and those in need of oxygen. In contrast, there was a significantly higher increase in the odds ratio of transportation difficulty in the target area than in the comparison area in Group 1 (odds ratio 1.393, 95% CI 1.314–1.477). This unfavorable change was more prominent in patients with respiratory diseases (odds ratio 2.767, 95% CI 2.349–3.260). The full estimation results with the coefficients (odds ratios) and confidence intervals of all covariates are reported in S1 Table.

**Table 3:** Multivariate regression analysis and stratified analysis of change in transportation time and number of transportation difficulty cases between before and after the first COVID-19 outbreak.

	Transportation time, min [Ordinary Least Squares]			Transportation difficulty, odds ratio [Logistic regression analysis]		
	Group 1 – Chiba	Group 2 – Kanagawa	Group 3 – Aichi	Group 1 – Chiba	Group 2 – Kanagawa	Group 3 – Aichi
**All cases (95% CI)**	0.214 (−1.364 to 1.792)	−2.016 (−3.631 to −0.400)	−0.606 (−1.114 to −0.098)	1.393 (1.314 to 1.477)	0.131 (0.110 to 0.157)	0.086 (0.066 to 0.112)
Mean of Dependent Variable	43.28	35.45	30.45	0.042	0.013	0.010
Observations	150856	186965	516656	150856	186965	516656
**Respiratory diseases (95% CI)**	3.378 (1.265 to 5.492)	−4.010 (−6.952 to −1.067)	−1.141 (−1.791 to −0.491)	2.767 (2.349 to 3.260)	0.105 (0.059 to 0.185)	0.075 (0.031 to 0.182)
Mean of Dependent Variable	42.27	34.96	30.36	0.034	0.013	0.010
Observations	13454	13640	50636	13454	12940	50428
**Demand for oxygen (95% CI)**	2.350 (−0.144 to 4.845)	−2.251 (−4.123 to −0.379)	−0.948 (−1.487 to −0.409)	1.829 (1.630 to 2.052)	0.180 (0.128 to 0.254)	0.119 (0.073 to 0.194)
Mean of Dependent Variable	44.28	35.6	30.33	0.065	0.014	0.009
Observations	23697	39040	108028	23697	36986	108028
**Severe (95% CI)**	1.573 (−0.798 to 3.945)	−1.440 (−2.959 to 0.079)	−0.419 (−0.745 to −0.092)	1.384 (1.130 to 1.696)	0.246 (0.132 to 0.457)	0.314 (0.139 to 0.708)
Mean of Dependent Variable	43.19	35.07	28.53	0.054	0.009	0.003
Observations	9270	14821	25691	9270	14499	25206
**Moderate (95% CI)**	0.751 (−1.325 to 2.828)	−2.450 (−4.332 to −0.569)	−0.971 (−1.497 to −0.445)	1.385 (1.277 to 1.501)	0.110 (0.086 to 0.141)	0.088 (0.061 to 0.127)
Mean of Dependent Variable	44.39	35.93	30.88	0.048	0.013	0.011
Observations	69278	101047	209290	69278	98028	209290
**Mild (95% CI)**	−0.414 (−0.961 to 0.132)	−1.718 (−3.223 to −0.214)	−0.352 (−0.940 to 0.236)	1.400 (1.274 to 1.539)	0.156 (0.116 to 0.209)	0.064 (0.040 to 0.100)
Mean of Dependent Variable	42.27	34.96	30.36	0.034	0.013	0.010
Observations	71491	67494	276347	71491	67317	276347
Month FIxed Effect	yes	yes	yes	yes	yes	yes
Fire Department Fixed Effect	yes	yes	yes	yes	yes	yes
Other Covariates	yes	yes	yes	yes	yes	yes

Note: We used ordinary least square for transportation time and logistic regression analysis for transportation difficulty.

Abbreviation: CI, confidence interval.

## Discussion

This retrospective observational study showed that the target areas of Group 2 and 3 showed significantly less worsening of transportation performance during the COVID-19 pandemic compared with the comparison areas. Compared to the comparison areas, the increase in transportation time in the target areas of Group 2 and 3 was 2 min and 40 seconds shorter, respectively. In contrast, transportation time increased in the target area of Group 1 as well as in the comparison area. Even in a population limited to patients with respiratory diseases or those receiving oxygen, the target areas of Group 2 and 3 showed favorable results. It should be noted that the dataset used in this study did not include post-transport patient outcomes, such as mortality and therefore, whether the observed differences in transportation time contributed to improved patient outcomes remains unclear. However, the study findings may provide important insights into the development of a resilient emergency transportation system during pandemics. To the best of our knowledge, this is the first study to analyze transportation time and difficulty during the COVID-19 pandemic in Japan compared with other cities using a large-scale database.

Few studies have been conducted on interventions that address transportation difficulties in Japan. Sato et al. reported that an increase in reimbursements for ambulance transportation acceptance was associated with decreased transportation difficulties [16]. Higashi et al. reported that the presence of the DEP Model contributed to reduced pre-hospital transportation time [15]. However, these studies were conducted before the COVID-19 pandemic, and it is unknown whether these effects remain during the COVID-19 pandemic. In Korea, early detection using expanded diagnostic tests and out-of-hospital treatment at therapeutic living centers was effective in countering the COVID-19 pandemic [20,21]. In contrast, Taiwan had successfully controlled COVID-19 through non-pharmaceutical interventions including finding, testing, tracing, isolating, and supporting (FTTIS) COVID-19 cases [22]. To our knowledge, no studies have verified the effectiveness of these countermeasures statistically. This study discusses interventions that can contribute to favorable transportation times and difficulties by comparing transportation results between areas with and without a DEP Model in Japan.

We speculate that the target areas of Groups 2 and 3 outperformed nearby areas, although this phenomenon was not observed in Group 1. First, Table 1 shows that large-scale hospitals are densely packed in the target areas of Groups 2 and 3 compared with Group 1. This could be attributed to the favorable results in Groups 2 and 3; namely, small-scale ER could not function effectively during the pandemic, and the importance of large-scale ER might become apparent during the pandemic.

The Shonan Kamakura General Hospital in the Group 2 target area built a makeshift building with 180 beds immediately next to the hospital in May 2020 to address the shortage of beds due to the COVID-19 pandemic. Similarly, Ichinomiyanishi Hospital in the Group 3 target area built a makeshift building with 25 beds immediately next to the hospital. These additional beds might have contributed to the favorable transportation results in Groups 2 and 3. One possible reason why the DEP system alone did not function effectively during the COVID-19 pandemic in Group 1 is that the primary bottleneck may have shifted from patient acceptance at the ED entrance to patient outflow. A previous study demonstrated that in the DEP system, dedicated emergency physicians can take care of emergency patients regardless of their condition or severity, resulting to increased capacity to accept a larger number of patients [15]. In other words, the DEP system may improve acceptance capacity at the ED entrance and as a result, contribute to shorter transportation times in the region. However, during the COVID-19 pandemic, patient outflow may have been obstructed by a relative shortage of inpatient beds and the advantages of the DEP system may have been offset.

Despite the development of vaccines and treatments for COVID-19, epidemics of COVID-19 still occur periodically. Furthermore, new infectious diseases with high infectivity may emerge and cause future pandemics. Then, the pre-hospital transportation problems will be encountered again. Based on this study, a temporary facility that accommodates patients with pandemic disease just next to a large-scale hospital and merges small-scale hospitals to large-scale hospitals may be beneficial in preparation for the next pandemic.

This study has some limitations. First, although we tried to minimize the bias caused by regional variations in COVID-19 prevalence, concerns remain that such variations may have affected the outcomes. Secondly, the data lacked post-transportation information. Therefore, it remains unclear whether changes in transportation outcomes, such as a 40-second reduction in transportation time, have a significant impact on patient outcomes. An association between ambulance offload time and 30-day mortality and ambulance re-attendance rates has been reported in patients with non-traumatic chest pain [23]. Likewise, total transport time may be associated with patient outcomes; however, further investigation is warranted. Third, we only analyzed pre-hospital data in Japan, and the results may not be generalizable to other countries where pre-hospital settings are different. Fourth, this was a retrospective observational study, and causation could not be determined.

## Conclusion

The DEP Model alone was not significantly associated with favorable transportation outcomes during the pandemic. The observed regional differences in transportation performance during the pandemic suggest that a variety of factors, such as inpatient bed availability in addition to the physicians’ capacity to accept and manage emergency patients, should be considered when preparing for future pandemics. Further studies are needed to evaluate the effectiveness and feasibility of these strategies.

## Supporting information

S1 Table Full estimation results for main analysis. (XLSX)

S1 Fig Kernel density plots of transportation time for each group before and after the COVID-19 pandemic. (PPTX)
