# Supplementary material for: Beyond the freedom to refuse patient: A retrospective comparative study of emergency transportation during the COVID-19 pandemic in Japan
Source: PLoS One. 2026 Jul 9;21(7):e0331535. doi: 10.1371/journal.pone.0331535 (PMC13349190; doi:10.1371/journal.pone.0331535)
Supplement: S1 Fig — (PPTX) [file pone.0331535.s002.pptx]

## Slide 1
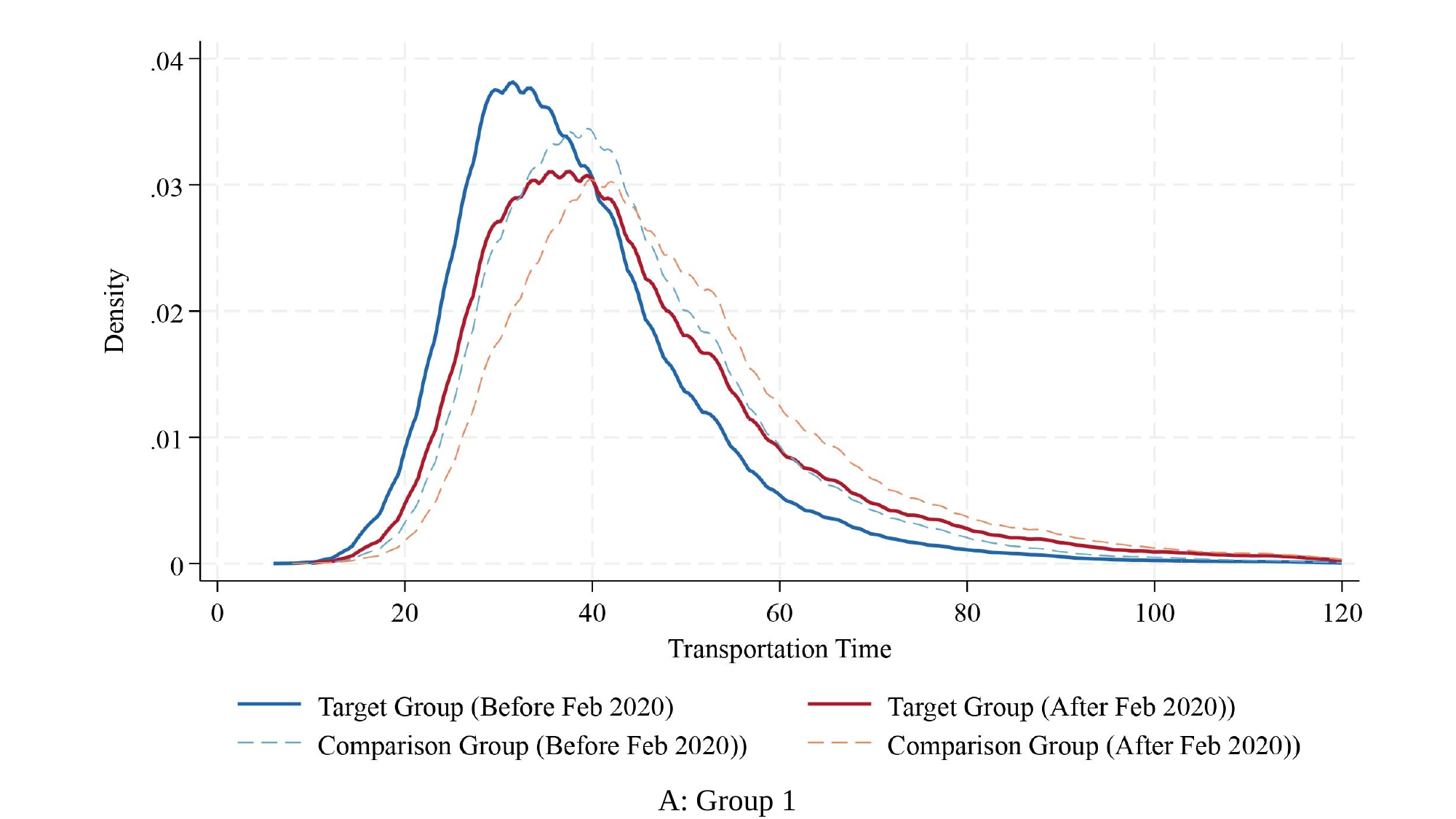

A: Group 1

## Slide 2
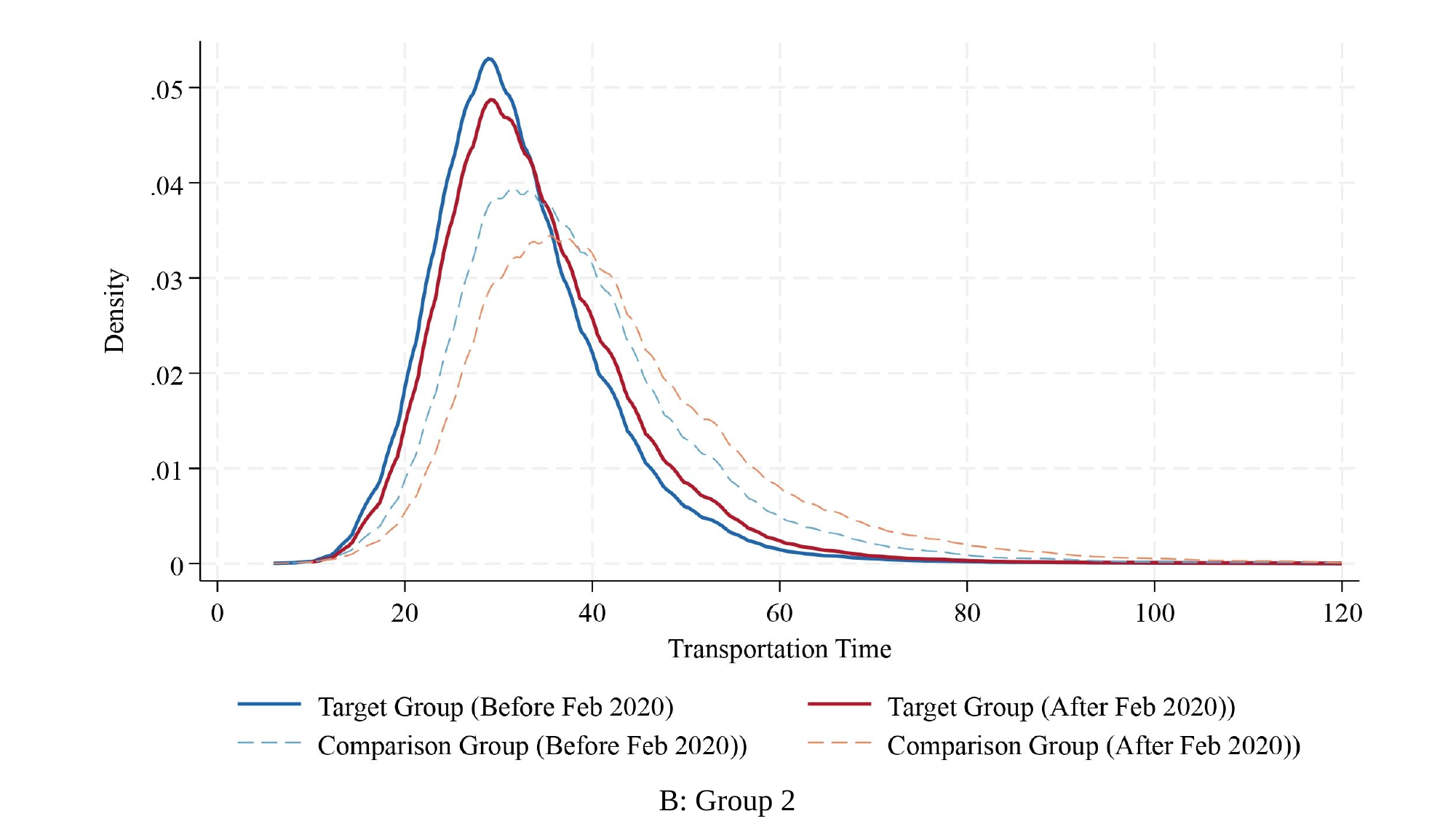

B: Group 2

## Slide 3
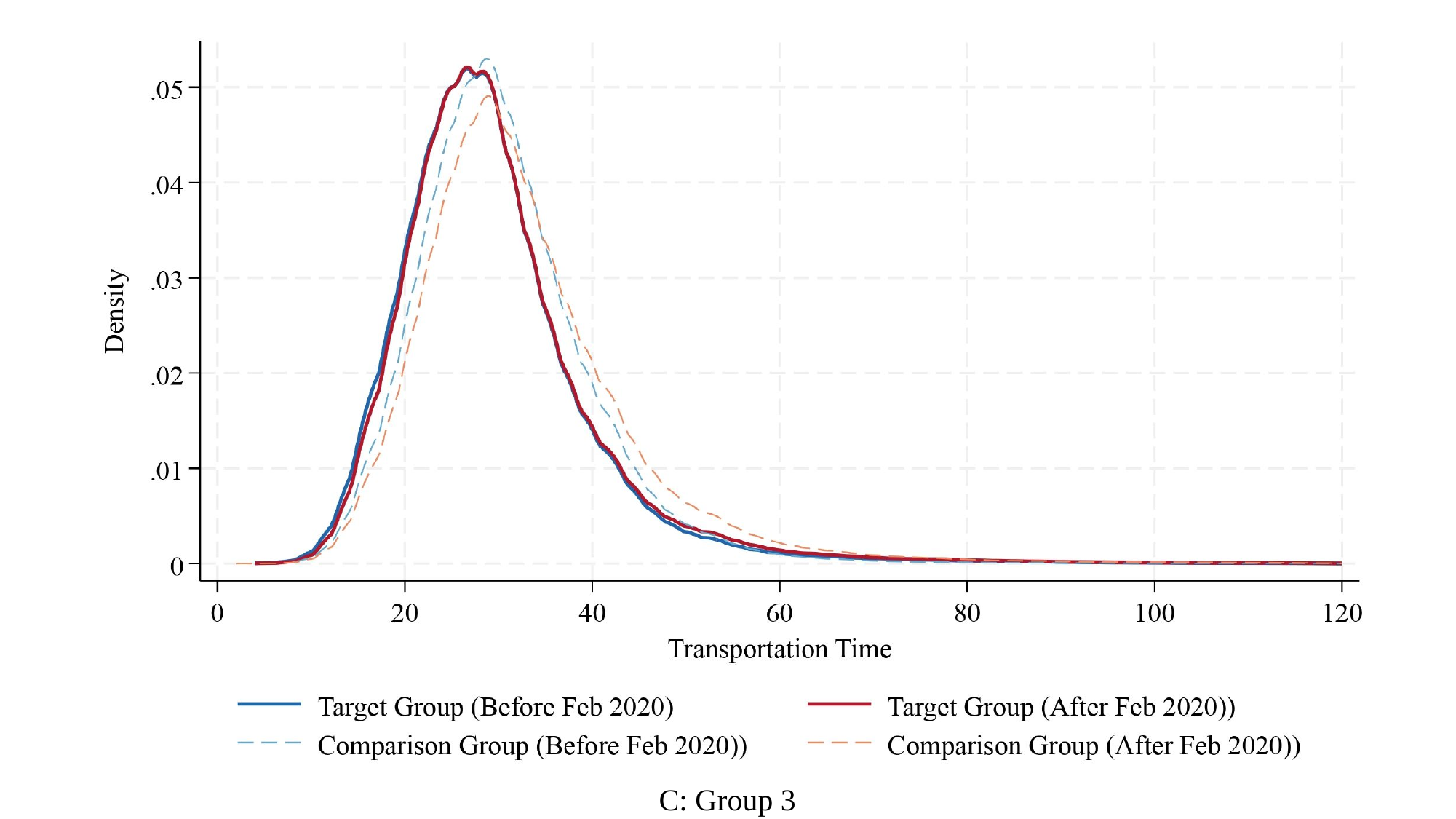

C: Group 3

## Slide 4
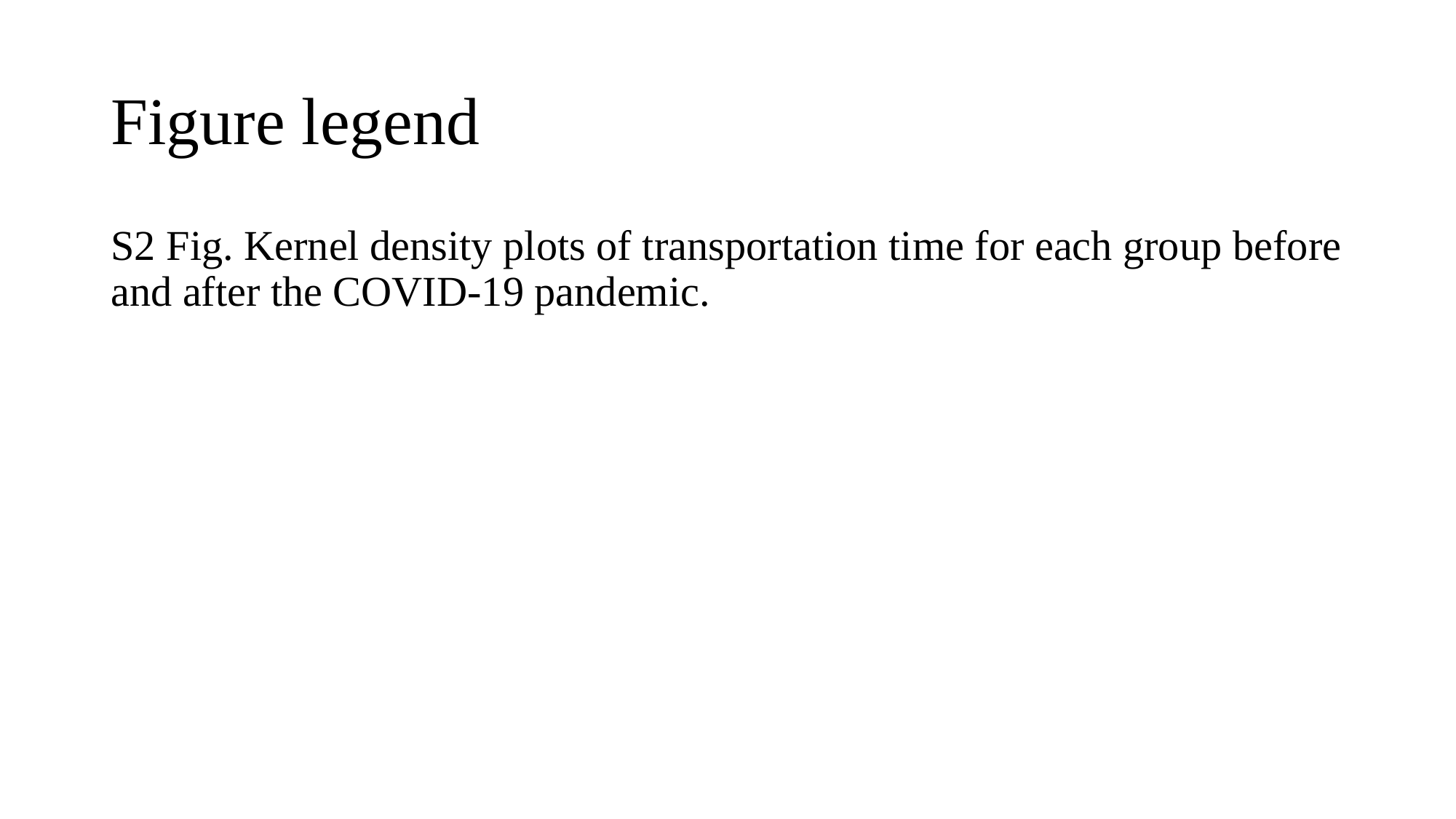

# Figure legend
S2 Fig. Kernel density plots of transportation time for each group before and after the COVID-19 pandemic.
